# 7-Benzene­sulfonamido-3-ethenyl-8-oxo-5-thia-1-aza­bicyclo­[4.2.0]oct-2-ene-2-carboxylic acid methanol solvate

**DOI:** 10.1107/S1600536809024726

**Published:** 2009-07-01

**Authors:** Irfana Mariam, Mehmet Akkurt, Shahzad Sharif, Syed Kamran Haider, Islam Ullah Khan

**Affiliations:** a Materials Chemistry Laboratory, Department of Chemistry, Government College University, Lahore 54000, Pakistan; b Department of Physics, Faculty of Arts and Sciences, Erciyes University, 38039 Kayseri, Turkey

## Abstract

In the title compound, C_15_H_14_N_2_O_5_S_2_·CH_4_O, the six-membered ring fused to the β-lactam unit adopts a twisted conformation. In the crystal structure, the component mol­ecules are linked into a three-dimensional framework through inter­molecular N—H⋯S,  N—H⋯O and O—H⋯O hydrogen bonds and C—H⋯O contacts.

## Related literature

For background to the use of the title compound in organic synthesis, see: Yamanaka *et al.* (1985 ▶). For ring puckering analysis, see: Cremer & Pople (1975 ▶). For reference structural data, see: Allen *et al.* (1987 ▶).
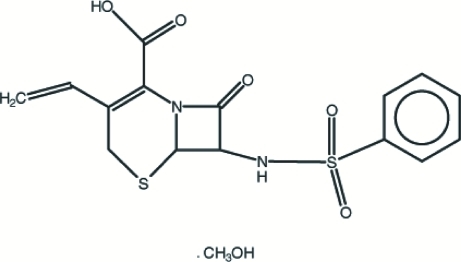


## Experimental

### 

#### Crystal data

C_15_H_14_N_2_O_5_S_2_·CH_4_O
*M* *_r_* = 398.46
Monoclinic, 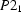

*a* = 12.0000 (2) Å
*b* = 6.0964 (8) Å
*c* = 13.602 (2) Å
β = 109.412 (7)°
*V* = 938.51 (19) Å^3^
*Z* = 2
Mo *K*α radiation
μ = 0.32 mm^−1^
*T* = 296 K
0.31 × 0.28 × 0.11 mm

#### Data collection

Bruker Kappa APEXII CCD area-detector diffractometer
Absorption correction: none
9995 measured reflections
3862 independent reflections
2296 reflections with *I* > 2σ(*I*)
*R*_int_ = 0.125

#### Refinement

*R*[*F* ^2^ > 2σ(*F* ^2^)] = 0.051
*wR*(*F* ^2^) = 0.120
*S* = 0.96
3862 reflections
238 parameters
1 restraint
H-atom parameters constrained
Δρ_max_ = 0.30 e Å^−3^
Δρ_min_ = −0.25 e Å^−3^
Absolute structure: Flack (1983 ▶), 1498 Freidel pairs
Flack parameter: 0.01 (10)

### 

Data collection: *APEX2* (Bruker, 2007 ▶); cell refinement: *SAINT* (Bruker, 2007 ▶); data reduction: *SAINT*; program(s) used to solve structure: *SHELXS97* (Sheldrick, 2008 ▶); program(s) used to refine structure: *SHELXL97* (Sheldrick, 2008 ▶); molecular graphics: *ORTEP-3 for Windows* (Farrugia, 1997 ▶); software used to prepare material for publication: *WinGX* (Farrugia, 1999 ▶) and *PLATON* (Spek, 2009 ▶).

## Supplementary Material

Crystal structure: contains datablocks global, I. DOI: 10.1107/S1600536809024726/hb5002sup1.cif

Structure factors: contains datablocks I. DOI: 10.1107/S1600536809024726/hb5002Isup2.hkl

Additional supplementary materials:  crystallographic information; 3D view; checkCIF report

## Figures and Tables

**Table 1:** Hydrogen-bond geometry (Å, °)

*D*—H⋯*A*	*D*—H	H⋯*A*	*D*⋯*A*	*D*—H⋯*A*
N1—H1⋯S2	0.86	2.76	3.111 (4)	106
N1—H1⋯O5^i^	0.86	2.30	2.888 (5)	126
O4—H4*A*⋯O6^ii^	0.82	1.76	2.575 (5)	171
O6—H6*A*⋯O3	0.82	2.00	2.799 (5)	166
C2—H2⋯O1	0.93	2.53	2.888 (7)	103
C7—H7⋯O1^iii^	0.98	2.34	3.037 (6)	127
C7—H7⋯O2	0.98	2.45	2.906 (6)	108
C12—H12*A*⋯O2^iv^	0.97	2.46	3.327 (6)	148
C13—H13⋯O4	0.93	2.44	2.982 (6)	117

Symmetry codes: (i) 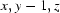; (ii) 
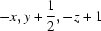
; (iii) 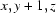; (iv) 
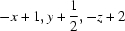
.

**Fig. 1.**
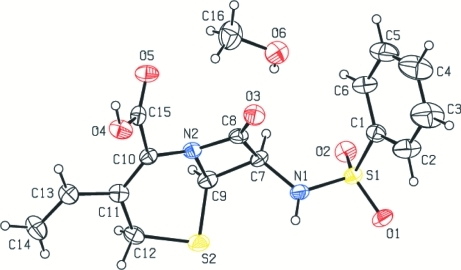
View of the title molecule with the atom numbering scheme. Displacement ellipsoids for non-H atoms are drawn at the 30% probability level.

**Fig. 2.**
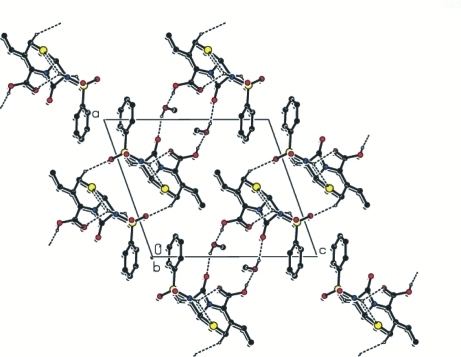
The packing and hydrogen bonding of (I) viewed down b axis. Hydrogen atoms not involved in the showed interactions have been omitted for clarity.

## supplementary crystallographic information

### Comment

(6*R*,7*R*)-7-Amino-3-ethenyl-8-oxo-5-thia-1-azabicyclo[4.2.0]oct-2-ene-2- carboxylic (7-AVCA) is an important intermediate for the synthesis of semi synthetic cephalosporins such as cefixime and cefdinir *etc*. These antibiotics are synthesized by the reaction of side chains such as amino thiazole thioesters with 7-AVCA (Yamanaka *et al.* 1985).

A view of compound (I) is shown in Fig. 1: its bond lengths and bond angles are within normal ranges (Allen *et al.*, 1987). In the main molecule of (I), the β-lactam unit (N2/C7—C9) and the six-membered ring fused to the β-lactam unit, (N2/S2/C9—C12) are puckered with the puckering parameters (Cremer & Pople, 1975): Q(2) = -0.102 (5) Å, and Q_T_ = 0.643 (4) Å, θ = 123.7 (4) °, φ = 197.2 (5) °, respectively.

In the crystal packing, intermolecular N—H···O, C—H···O contacts and O—H···O hydrogen bonds stabilize crystal structure (Table 1, Fig. 2).

### Experimental

7-AVCA (1 g, 4.4 mmol) was dissolved in distilled water (15 ml) in a round bottom flask (50 ml). 3*M* Na_2_CO_3_ solution was used to maintain the solution at pH at 8–9, then benzene sulfonyl chloride (1.1 g, 4.4 mmol) was added to the solution and stirred at room temperature until all the benzene sulfonyl chloride was consumed. When reaction competed, the pH was adjusted 1–2, using 3 N HCl. The precipitate obtained was filtered, washed with distilled water, dried and recrystalized in methanol and ethyl acetate, from which light yellow prisms of (I) appeared after three days.

### Refinement

All H atoms were located geometrically and treated as riding with C—H = 0.93 Å (aromatic), 0.97 Å (methylene) or 0.98 Å (methine), N—H = 0.86 Å and O—H = 0.82 Å with *U*_iso_(H) = 1.2 or 1.5*U*_eq_(C, N, O). The Friedel pairs were not merged and the Flack parameter (Flack, 1983) [with 1498 Freidel pairs] yielded an indeterminate value with large uncertainty [0.01 (10)]. The absolute configuration was established from the starting material.

### Crystal data

**Table:**

C_15_H_14_N_2_O_5_S_2_·CH_4_O	*F*(000) = 416
*M**_r_* = 398.46	*D*_x_ = 1.410 Mg m^−^^3^
Monoclinic, *P*2_1_	Mo *K*α radiation, λ = 0.71073 Å
Hall symbol: P 2yb	Cell parameters from 1813 reflections
*a* = 12.0000 (2) Å	θ = 2.8–21.7°
*b* = 6.0964 (8) Å	µ = 0.32 mm^−^^1^
*c* = 13.602 (2) Å	*T* = 296 K
β = 109.412 (7)°	Prism, light yellow
*V* = 938.51 (19) Å^3^	0.31 × 0.28 × 0.11 mm
*Z* = 2	

### Data collection

**Table:**

Bruker Kappa APEXII CCD area-detector diffractometer	2296 reflections with *I* > 2σ(*I*)
Radiation source: sealed tube	*R*_int_ = 0.125
graphite	θ_max_ = 27.5°, θ_min_ = 2.8°
φ and ω scans	*h* = −15→15
9995 measured reflections	*k* = −7→7
3862 independent reflections	*l* = −17→17

### Refinement

**Table:**

Refinement on *F*^2^	Secondary atom site location: difference Fourier map
Least-squares matrix: full	Hydrogen site location: inferred from neighbouring sites
*R*[*F*^2^ > 2σ(*F*^2^)] = 0.051	H-atom parameters constrained
*wR*(*F*^2^) = 0.120	*w* = 1/[σ^2^(*F*_o_^2^)], where *P* = (*F*_o_^2^ + 2*F*_c_^2^)/3
*S* = 0.96	(Δ/σ)_max_ < 0.001
3862 reflections	Δρ_max_ = 0.30 e Å^−^^3^
238 parameters	Δρ_min_ = −0.25 e Å^−^^3^
1 restraint	Absolute structure: Flack (1983), 1498 Freidel pairs
Primary atom site location: structure-invariant direct methods	Flack parameter: 0.01 (10)

### Special details

**Table:**

Geometry. Bond distances, angles *etc*. have been calculated using the rounded fractional coordinates. All su's are estimated from the variances of the (full) variance-covariance matrix. The cell e.s.d.'s are taken into account in the estimation of distances, angles and torsion angles
Refinement. Refinement on *F*^2^ for ALL reflections except those flagged by the user for potential systematic errors. Weighted *R*-factors *wR* and all goodnesses of fit *S* are based on *F*^2^, conventional *R*-factors *R* are based on *F*, with *F* set to zero for negative *F*^2^. The observed criterion of *F*^2^ > σ(*F*^2^) is used only for calculating -*R*-factor-obs *etc*. and is not relevant to the choice of reflections for refinement. *R*-factors based on *F*^2^ are statistically about twice as large as those based on *F*, and *R*-factors based on ALL data will be even larger.

### Fractional atomic coordinates and isotropic or equivalent isotropic displacement parameters (Å^2^)

**Table:**

	*x*	*y*	*z*	*U*_iso_*/*U*_eq_	
S1	0.24181 (10)	0.36361 (18)	0.94436 (9)	0.0411 (3)	
S2	0.50199 (10)	0.6029 (2)	0.77686 (11)	0.0521 (4)	
O1	0.2562 (3)	0.1318 (5)	0.9415 (3)	0.0542 (11)	
O2	0.2889 (3)	0.4810 (6)	1.0402 (2)	0.0517 (11)	
O3	0.1400 (3)	0.8330 (5)	0.7110 (3)	0.0549 (11)	
O4	0.2503 (3)	1.1724 (5)	0.5108 (2)	0.0525 (13)	
O5	0.2210 (3)	1.2961 (5)	0.6536 (3)	0.0515 (11)	
N1	0.3034 (3)	0.4643 (6)	0.8640 (3)	0.0429 (12)	
N2	0.3414 (3)	0.9197 (6)	0.7515 (3)	0.0395 (11)	
C1	0.0903 (4)	0.4207 (8)	0.8928 (4)	0.0452 (16)	
C2	0.0153 (5)	0.2642 (10)	0.8338 (5)	0.072 (2)	
C3	−0.1051 (6)	0.3020 (13)	0.7971 (6)	0.106 (3)	
C4	−0.1480 (5)	0.4972 (15)	0.8162 (6)	0.106 (4)	
C5	−0.0732 (6)	0.6566 (11)	0.8740 (6)	0.083 (3)	
C6	0.0463 (5)	0.6175 (9)	0.9130 (4)	0.0605 (19)	
C7	0.3184 (4)	0.7005 (7)	0.8608 (4)	0.0382 (14)	
C8	0.2425 (4)	0.8224 (7)	0.7622 (4)	0.0395 (16)	
C9	0.4254 (4)	0.7890 (7)	0.8338 (3)	0.0414 (14)	
C10	0.3684 (4)	1.0245 (7)	0.6711 (3)	0.0363 (12)	
C11	0.4728 (4)	0.9955 (8)	0.6583 (4)	0.0430 (16)	
C12	0.5600 (4)	0.8213 (8)	0.7183 (4)	0.0477 (16)	
C13	0.5131 (4)	1.1393 (8)	0.5911 (4)	0.0505 (17)	
C14	0.6165 (5)	1.1290 (11)	0.5780 (4)	0.066 (2)	
C15	0.2738 (4)	1.1796 (7)	0.6118 (4)	0.0396 (16)	
O6	−0.0926 (3)	0.9464 (6)	0.5948 (3)	0.0612 (13)	
C16	−0.0774 (5)	1.1397 (11)	0.5436 (5)	0.087 (3)	
H1	0.32650	0.37930	0.82420	0.0510*	
H2	0.04550	0.13290	0.81850	0.0860*	
H3	−0.15650	0.19400	0.75960	0.1280*	
H4	−0.22890	0.52390	0.79000	0.1270*	
H4A	0.19880	1.26240	0.48270	0.0790*	
H5	−0.10350	0.79010	0.88660	0.1000*	
H6	0.09720	0.72360	0.95290	0.0730*	
H7	0.31290	0.77040	0.92390	0.0450*	
H9	0.47870	0.87900	0.88940	0.0490*	
H12A	0.62240	0.89400	0.77300	0.0570*	
H12B	0.59600	0.75590	0.67110	0.0570*	
H13	0.46110	1.24730	0.55440	0.0600*	
H14A	0.67120	1.02350	0.61320	0.0790*	
H14B	0.63520	1.22730	0.53350	0.0790*	
H6A	−0.02910	0.90940	0.63720	0.0920*	
H16A	−0.14900	1.22400	0.52400	0.1300*	
H16B	−0.05870	1.10180	0.48230	0.1300*	
H16C	−0.01400	1.22470	0.58960	0.1300*	

### Atomic displacement parameters (Å^2^)

**Table:**

	*U*^11^	*U*^22^	*U*^33^	*U*^12^	*U*^13^	*U*^23^
S1	0.0419 (6)	0.0356 (6)	0.0425 (6)	0.0064 (5)	0.0098 (5)	0.0033 (6)
S2	0.0394 (6)	0.0459 (7)	0.0678 (9)	0.0128 (6)	0.0135 (6)	0.0032 (7)
O1	0.061 (2)	0.0331 (18)	0.068 (2)	0.0099 (15)	0.0208 (18)	0.0053 (17)
O2	0.057 (2)	0.056 (2)	0.0372 (19)	0.0017 (16)	0.0091 (16)	0.0000 (16)
O3	0.0350 (16)	0.061 (2)	0.061 (2)	0.0070 (16)	0.0055 (16)	0.0100 (19)
O4	0.0470 (19)	0.068 (3)	0.0376 (19)	0.0172 (15)	0.0074 (16)	0.0061 (17)
O5	0.0476 (18)	0.056 (2)	0.046 (2)	0.0181 (15)	0.0092 (16)	−0.0005 (16)
N1	0.051 (2)	0.035 (2)	0.047 (2)	0.0085 (17)	0.022 (2)	−0.0006 (18)
N2	0.0324 (18)	0.040 (2)	0.042 (2)	0.0113 (15)	0.0070 (16)	0.0087 (17)
C1	0.040 (2)	0.048 (3)	0.046 (3)	0.006 (2)	0.012 (2)	0.002 (2)
C2	0.055 (3)	0.066 (4)	0.083 (4)	0.011 (3)	0.006 (3)	−0.023 (3)
C3	0.053 (4)	0.117 (6)	0.124 (7)	0.005 (4)	−0.004 (4)	−0.036 (5)
C4	0.041 (4)	0.141 (7)	0.115 (7)	0.032 (4)	−0.001 (4)	−0.008 (6)
C5	0.074 (4)	0.088 (5)	0.091 (5)	0.035 (4)	0.032 (4)	0.003 (4)
C6	0.064 (3)	0.053 (3)	0.063 (4)	0.019 (3)	0.019 (3)	−0.004 (3)
C7	0.040 (2)	0.029 (2)	0.042 (3)	0.0047 (18)	0.009 (2)	0.003 (2)
C8	0.031 (2)	0.038 (3)	0.046 (3)	0.0073 (18)	0.0081 (19)	−0.003 (2)
C9	0.037 (2)	0.042 (3)	0.037 (2)	0.0061 (19)	0.0012 (19)	0.002 (2)
C10	0.034 (2)	0.034 (2)	0.036 (2)	0.0026 (17)	0.0050 (19)	0.0001 (19)
C11	0.039 (2)	0.048 (3)	0.037 (3)	0.003 (2)	0.006 (2)	−0.003 (2)
C12	0.035 (2)	0.058 (3)	0.048 (3)	0.006 (2)	0.011 (2)	−0.001 (2)
C13	0.047 (3)	0.058 (3)	0.046 (3)	0.003 (2)	0.015 (2)	0.004 (3)
C14	0.047 (3)	0.091 (4)	0.061 (4)	0.002 (3)	0.021 (3)	0.012 (4)
C15	0.037 (2)	0.040 (3)	0.039 (3)	−0.0050 (19)	0.009 (2)	0.007 (2)
O6	0.0373 (18)	0.066 (2)	0.064 (3)	−0.0017 (17)	−0.0051 (17)	−0.0037 (19)
C16	0.067 (4)	0.086 (5)	0.095 (5)	−0.017 (4)	0.010 (4)	0.005 (4)

### Geometric parameters (Å, °)

**Table:**

S1—O1	1.426 (3)	C7—C8	1.539 (7)
S1—O2	1.429 (3)	C7—C9	1.545 (7)
S1—N1	1.629 (4)	C10—C11	1.333 (7)
S1—C1	1.752 (5)	C10—C15	1.492 (7)
S2—C9	1.790 (5)	C11—C12	1.524 (7)
S2—C12	1.806 (5)	C11—C13	1.460 (7)
O3—C8	1.197 (6)	C13—C14	1.312 (8)
O4—C15	1.308 (6)	C2—H2	0.9300
O5—C15	1.212 (6)	C3—H3	0.9300
O4—H4A	0.8200	C4—H4	0.9300
O6—C16	1.411 (8)	C5—H5	0.9300
O6—H6A	0.8200	C6—H6	0.9300
N1—C7	1.454 (6)	C7—H7	0.9800
N2—C8	1.377 (6)	C9—H9	0.9800
N2—C10	1.395 (6)	C12—H12B	0.9700
N2—C9	1.468 (6)	C12—H12A	0.9700
N1—H1	0.8600	C13—H13	0.9300
C1—C6	1.375 (7)	C14—H14B	0.9300
C1—C2	1.373 (8)	C14—H14A	0.9300
C2—C3	1.382 (10)	C16—H16A	0.9600
C3—C4	1.356 (12)	C16—H16B	0.9600
C4—C5	1.378 (11)	C16—H16C	0.9600
C5—C6	1.374 (10)		
			
S2···N1	3.111 (4)	C16···O5^v^	3.388 (7)
S2···H1	2.7600	C16···O3	3.397 (7)
O1···C7^i^	3.037 (6)	C1···H5^vii^	3.0600
O1···C8^i^	3.044 (6)	C11···H14B^viii^	2.9900
O2···C12^ii^	3.327 (6)	C12···H14A	2.5700
O3···O5	3.167 (5)	C12···H4^ix^	3.0000
O3···N1	3.243 (5)	C14···H3^x^	3.0300
O3···C15	3.210 (6)	C14···H12A	2.9900
O3···O6	2.799 (5)	C14···H13^viii^	2.9000
O3···C16	3.397 (7)	C14···H12B	2.6500
O4···O6^iii^	2.575 (5)	C15···H1^iv^	3.0000
O4···C13	2.982 (6)	C15···H13	2.6500
O5···N2	2.801 (5)	C16···H4A^v^	2.6800
O5···C8	3.215 (6)	H1···S2	2.7600
O5···O3	3.167 (5)	H1···C15^i^	3.0000
O5···C16^iii^	3.388 (7)	H1···O5^i^	2.3000
O5···N1^iv^	2.888 (5)	H2···O1	2.5300
O6···C15^v^	3.348 (6)	H2···O3^i^	2.8100
O6···O4^v^	2.575 (5)	H3···H14A^xi^	2.5700
O6···O3	2.799 (5)	H3···C14^xi^	3.0300
O1···H7^i^	2.3400	H4···C12^xii^	3.0000
O1···H2	2.5300	H4A···O6^iii^	1.7600
O2···H12A^ii^	2.4600	H4A···C16^iii^	2.6800
O2···H6	2.6600	H4A···H6A^iii^	2.3200
O2···H9^ii^	2.7000	H5···C1^xiii^	3.0600
O2···H7	2.4500	H6···O2	2.6600
O3···H2^iv^	2.8100	H6A···O3	2.0000
O3···H16B^v^	2.8500	H6A···H4A^v^	2.3200
O3···H6A	2.0000	H7···O1^iv^	2.3400
O4···H13	2.4400	H7···O2	2.4500
O5···H1^iv^	2.3000	H9···O2^vi^	2.7000
O5···H16C	2.7000	H12A···C14	2.9900
O5···H16B^iii^	2.8800	H12A···H14A	2.5600
O6···H4A^v^	1.7600	H12A···O2^vi^	2.4600
N1···S2	3.111 (4)	H12B···C14	2.6500
N1···O3	3.243 (5)	H12B···H14A	2.1400
N1···N2	3.274 (5)	H13···C14^xiv^	2.9000
N1···O5^i^	2.888 (5)	H13···C15	2.6500
N2···O5	2.801 (5)	H13···O4	2.4400
N2···N1	3.274 (5)	H14A···H3^x^	2.5700
C7···O1^iv^	3.037 (6)	H14A···C12	2.5700
C8···O5	3.215 (6)	H14A···H12A	2.5600
C8···O1^iv^	3.044 (6)	H14A···H12B	2.1400
C12···O2^vi^	3.327 (6)	H14B···C11^xiv^	2.9900
C13···O4	2.982 (6)	H16B···O3^iii^	2.8500
C15···O6^iii^	3.348 (6)	H16B···O5^v^	2.8800
C15···O3	3.210 (6)	H16C···O5	2.7000
			
O1—S1—O2	120.4 (2)	S2—C12—C11	117.0 (4)
O1—S1—N1	105.6 (2)	C11—C13—C14	125.5 (5)
O1—S1—C1	107.8 (2)	O4—C15—O5	123.3 (5)
O2—S1—N1	107.1 (2)	O5—C15—C10	122.7 (5)
O2—S1—C1	108.0 (2)	O4—C15—C10	114.0 (4)
N1—S1—C1	107.4 (2)	C1—C2—H2	120.00
C9—S2—C12	93.0 (2)	C3—C2—H2	120.00
C15—O4—H4A	109.00	C2—C3—H3	120.00
C16—O6—H6A	110.00	C4—C3—H3	120.00
S1—N1—C7	118.8 (3)	C5—C4—H4	120.00
C9—N2—C10	124.6 (4)	C3—C4—H4	120.00
C8—N2—C9	94.8 (4)	C4—C5—H5	120.00
C8—N2—C10	135.5 (4)	C6—C5—H5	120.00
C7—N1—H1	121.00	C5—C6—H6	120.00
S1—N1—H1	121.00	C1—C6—H6	120.00
C2—C1—C6	120.2 (5)	N1—C7—H7	111.00
S1—C1—C2	119.2 (4)	C9—C7—H7	111.00
S1—C1—C6	120.6 (4)	C8—C7—H7	111.00
C1—C2—C3	119.9 (6)	N2—C9—H9	113.00
C2—C3—C4	119.7 (7)	C7—C9—H9	113.00
C3—C4—C5	120.8 (7)	S2—C9—H9	113.00
C4—C5—C6	119.7 (6)	S2—C12—H12B	108.00
C1—C6—C5	119.7 (5)	C11—C12—H12A	108.00
N1—C7—C9	118.3 (4)	S2—C12—H12A	108.00
N1—C7—C8	117.5 (4)	H12A—C12—H12B	107.00
C8—C7—C9	85.5 (3)	C11—C12—H12B	108.00
O3—C8—C7	136.8 (5)	C11—C13—H13	117.00
O3—C8—N2	132.1 (5)	C14—C13—H13	117.00
N2—C8—C7	91.1 (4)	C13—C14—H14B	120.00
S2—C9—C7	117.9 (3)	H14A—C14—H14B	120.00
N2—C9—C7	87.5 (3)	C13—C14—H14A	120.00
S2—C9—N2	109.6 (3)	O6—C16—H16A	109.00
N2—C10—C11	121.0 (4)	O6—C16—H16B	109.00
N2—C10—C15	112.4 (4)	O6—C16—H16C	109.00
C11—C10—C15	126.4 (4)	H16A—C16—H16B	109.00
C10—C11—C13	121.6 (4)	H16A—C16—H16C	110.00
C10—C11—C12	122.1 (4)	H16B—C16—H16C	109.00
C12—C11—C13	116.3 (4)		
			
O1—S1—N1—C7	−171.2 (4)	C6—C1—C2—C3	−1.9 (9)
O2—S1—N1—C7	−41.7 (4)	S1—C1—C2—C3	176.6 (5)
C1—S1—N1—C7	74.0 (4)	C2—C1—C6—C5	0.1 (9)
O1—S1—C1—C2	−17.8 (5)	C1—C2—C3—C4	2.6 (11)
O2—S1—C1—C2	−149.4 (4)	C2—C3—C4—C5	−1.6 (12)
N1—S1—C1—C2	95.5 (5)	C3—C4—C5—C6	−0.1 (11)
O1—S1—C1—C6	160.6 (4)	C4—C5—C6—C1	0.9 (10)
O2—S1—C1—C6	29.1 (5)	C8—C7—C9—N2	7.4 (3)
N1—S1—C1—C6	−86.0 (5)	N1—C7—C8—N2	−127.6 (4)
C12—S2—C9—N2	58.9 (3)	C9—C7—C8—O3	171.1 (6)
C12—S2—C9—C7	156.7 (4)	N1—C7—C8—O3	51.4 (8)
C9—S2—C12—C11	−48.2 (4)	N1—C7—C9—S2	15.4 (6)
S1—N1—C7—C9	148.9 (3)	N1—C7—C9—N2	126.3 (4)
S1—N1—C7—C8	−110.7 (4)	C8—C7—C9—S2	−103.5 (3)
C10—N2—C8—O3	−17.0 (9)	C9—C7—C8—N2	−7.9 (3)
C9—N2—C8—C7	8.3 (3)	N2—C10—C11—C12	9.9 (7)
C9—N2—C10—C15	−168.8 (4)	N2—C10—C11—C13	−165.8 (4)
C8—N2—C10—C15	43.6 (7)	C15—C10—C11—C13	8.4 (8)
C9—N2—C8—O3	−170.7 (5)	N2—C10—C15—O4	−138.0 (4)
C10—N2—C9—S2	−47.4 (5)	C15—C10—C11—C12	−175.9 (4)
C8—N2—C9—C7	−8.3 (3)	C11—C10—C15—O4	47.3 (6)
C10—N2—C9—C7	−166.2 (4)	C11—C10—C15—O5	−135.0 (5)
C10—N2—C8—C7	162.1 (5)	N2—C10—C15—O5	39.6 (6)
C9—N2—C10—C11	6.2 (7)	C10—C11—C12—S2	18.3 (6)
C8—N2—C10—C11	−141.5 (5)	C10—C11—C13—C14	176.6 (5)
C8—N2—C9—S2	110.5 (3)	C12—C11—C13—C14	0.6 (8)
S1—C1—C6—C5	−178.3 (5)	C13—C11—C12—S2	−165.8 (4)

Symmetry codes: (i) *x*, *y*−1, *z*; (ii) −*x*+1, *y*−1/2, −*z*+2; (iii) −*x*, *y*+1/2, −*z*+1; (iv) *x*, *y*+1, *z*; (v) −*x*, *y*−1/2, −*z*+1; (vi) −*x*+1, *y*+1/2, −*z*+2; (vii) −*x*, *y*−1/2, −*z*+2; (viii) −*x*+1, *y*−1/2, −*z*+1; (ix) *x*+1, *y*, *z*; (x) *x*+1, *y*+1, *z*; (xi) *x*−1, *y*−1, *z*; (xii) *x*−1, *y*, *z*; (xiii) −*x*, *y*+1/2, −*z*+2; (xiv) −*x*+1, *y*+1/2, −*z*+1.

### Hydrogen-bond geometry (Å, °)

**Table:**

*D*—H···*A*	*D*—H	H···*A*	*D*···*A*	*D*—H···*A*
N1—H1···S2	0.86	2.76	3.111 (4)	106
N1—H1···O5^i^	0.86	2.30	2.888 (5)	126
O4—H4A···O6^iii^	0.82	1.76	2.575 (5)	171
O6—H6A···O3	0.82	2.00	2.799 (5)	166
C2—H2···O1	0.93	2.53	2.888 (7)	103
C7—H7···O1^iv^	0.98	2.34	3.037 (6)	127
C7—H7···O2	0.98	2.45	2.906 (6)	108
C12—H12A···O2^vi^	0.97	2.46	3.327 (6)	148
C13—H13···O4	0.93	2.44	2.982 (6)	117

Symmetry codes: (i) *x*, *y*−1, *z*; (iii) −*x*, *y*+1/2, −*z*+1; (iv) *x*, *y*+1, *z*; (vi) −*x*+1, *y*+1/2, −*z*+2.
